# Minerals and atmosphere coevolve in the Earth’s history

**DOI:** 10.1093/nsr/nwag358

**Published:** 2026-06-12

**Authors:** Sergey V Krivovichev

**Affiliations:** Kola Science Centre, Russian Academy of Sciences; and Institute of Earth Sciences, St. Petersburg State University, Russia

Planetary evolution is one of the most mysterious issues in modern geology and geochemistry, because it concerns humans, that is, ourselves as a part of the general cosmic system of existence. It seems quite obvious that the mineral world, as the basis of the solid Earth, was also a subject to evolution with the passage of geological time. At the same time, apart from the early Soviet works in this field [1], the theory of mineral evolution took shape as a separate branch of mineralogy and geochemistry relatively recently [2]. It is now an indisputable fact that the mineral world developed on a par with the biological world, although the mechanisms of evolution of these two worlds are undoubtedly different.

The history of the Earth as a planet is accompanied by both an increase in mineral diversity (measured as the number of different mineral species and groups) and its structural and chemical complexity [3]. Mineral evolution is a sensitive indicator of global planetary processes: plate tectonics and revolutionary changes in living organisms are reflected in the composition and diversity of mineral associations, both at the local level and at the level of mineral deposits. Intuitively, this seems quite plausible, but how can one show this with specific scientific data? The Earth is constantly changing and often erases traces of past geospheres, so that we have only residual evidence, which may not be enough for reliable paleomineralogical reconstructions.

In a recent paper, Li *et al.* [4] convincingly show that analyzing mineral evolution at the available data level using deep learning techniques allows them to establish quantitative correlation between the development of the mineral world and the history of atmospheric oxygen. As an object of the study, Li *et al.* took manganese minerals, which possess an amazing variety due to a number of circumstances. First, in geochemical environments, manganese is redox-sensitive and can have three oxidation states: +2, +3 and +4, and in some minerals several oxidation states can be present simultaneously (as, for example, in armbrusterite, K_5_Na_6_Mn^3+^Mn^2+^_14_[Si_9_O_22_]_4_(OH)_10_·4H_2_O [5]; Fig. 1). Second, different manganese ions have different electronic and chemical properties: while Mn^2+^ exhibits both lithophilic and chalcophilic nature (about two dozen Mn^2+^ sulfide mineral species are currently known), Mn^3+^ and Mn^4+^ are strictly lithophilic. Third, manganese minerals are distributed in a wide variety of geological settings, from the Arctic alkaline complexes to the Kalahari Desert and the bottom of the World Ocean.

**Figure 1.**
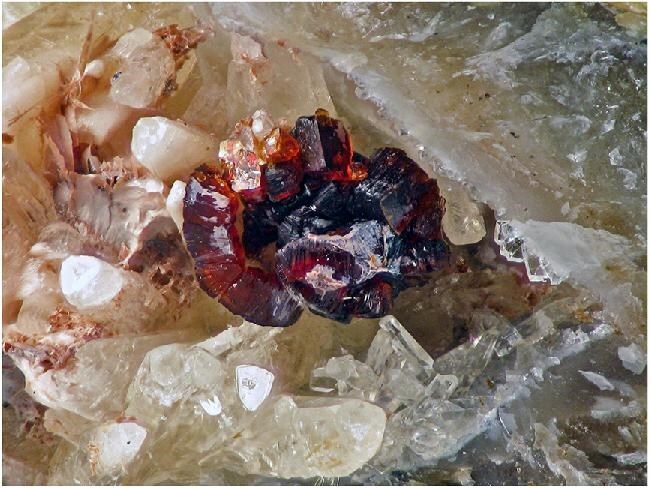
Reddish-brown plates of armbrusterite, K_5_Na_6_Mn^3+^Mn^2+^_14_[Si_9_O_22_]_4_(OH)_10_·4H_2_O, a mixed-valent Mn mineral formed ca. 0.4 Ga in hydrothermal veins of the Khibiny alkaline complex (Kola peninsula, Russia). The field of view is ~3 × 2.5 mm^2^.

In 2022, Hummer *et al.* [6] demonstrated that, during the last billion years, the average oxidation state of crustal Mn occurrences evolved with atmospheric oxygenation with a time lag of ~66 million years needed to equilibrate the Earth surface to atmospheric oxygen fugacity. Based on this work and going further, Li *et al.* extended their analysis using global Mn mineral dataset with 25 dimensions to build a model that links mineral evolution and the evolving redox state of our planet.

This work demonstrates the power and remarkable perspectives of mineral informatics [7], the emerging field of research that explores mineralogical big data with modern information techniques in order to find hidden patterns in deep-time evolution of our planet captured in a beautiful world of minerals.

The model proposed by the authors undoubtedly works, but, as in many other cases of models obtained using big data, there are still open questions related to the influence of certain factors on the effectiveness of the model. The choice of input data for the model is not always unambiguous and requires a deep understanding of the crystal-chemical properties of minerals. For example, the model by Li *et al.* does not use the manganese coordination number (which has a tremendous influence on crystal energetics), and symmetry was taken into account by belonging to a certain crystal system only, and not using quantitative indicators. Further prospects of the method developed by the authors may lie in the search for optimal mineral parameters for the input model, including thermodynamic characteristics, stability constants and numerical parameters of coordination and symmetry.

Speaking more broadly, the international mineral informatics community should develop unified dataset requirements for such models that will identify key parameters that form the basis of effectiveness in understanding certain features of the past of our planet and, possibly, other planets in the Solar System.

## References

[1] Zhabin AG . Dokl Earth Sci Sect 1981; 247: 142–4.

[2] Hazen RM, Papineau D, Bleeker W et al. Am Mineral 2008; 93: 1693–720. 10.2138/am.2008.2955

[3] Krivovichev SV, Krivovichev VG, Hazen RM. Eur J Mineral 2018; 30: 231–6. 10.1127/ejm/2018/0030-2694

[4] Li Y, Zhuang Z, Xu X et al. Natl Sci Rev 2026; 13: nwag230. 10.1093/nsr/nwag230 42282936 PMC13251890

[5] Yakovenchuk VN, Krivovichev SV, Pakhomovsky YA et al. Am Mineral 2007; 92: 416–23. 10.2138/am.2007.2200

[6] Hummer DR, Golden JJ, Hystad G et al. Nat Comm 2022; 13: 960. 10.1038/s41467-022-28589-x PMC8857192 35181670

[7] Prabhu A, Morrison SM, Fox P et al. Am Mineral 2023; 108: 1242–57. 10.2138/am-2022-8613

